# The potential impacts of micro-and-nano plastics on various organ systems in humans

**DOI:** 10.1016/j.ebiom.2023.104901

**Published:** 2023-12-06

**Authors:** Nurshad Ali, Jenny Katsouli, Emma L. Marczylo, Timothy W. Gant, Stephanie Wright, Jorge Bernardino de la Serna

**Affiliations:** a National Heart and Lung Institute, Imperial College London, Sir Alexander Fleming Building, London, SW7 2AZ, UK; b Department of Biochemistry and Molecular Biology, Shahjalal University of Science and Technology, Sylhet, 3114, Bangladesh; c MRC Centre for Environment and Health, School of Public Health, Imperial College London, London, UK; d Toxicology Department, Radiation, Chemical and Environmental Hazards, UK Health Security Agency, Harwell Campus, Chilton, Oxfordshire, OX11 0RQ, UK

**Keywords:** Microplastic, Nanoplastics, Human exposure, Organ system, Health effects, Toxicity

## Abstract

Humans are exposed to micro-and-nano plastics (MNPs) through various routes, but the adverse health effects of MNPs on different organ systems are not yet fully understood. This review aims to provide an overview of the potential impacts of MNPs on various organ systems and identify knowledge gaps in current research. The summarized results suggest that exposure to MNPs can lead to health effects through oxidative stress, inflammation, immune dysfunction, altered biochemical and energy metabolism, impaired cell proliferation, disrupted microbial metabolic pathways, abnormal organ development, and carcinogenicity. There is limited human data on the health effects of MNPs, despite evidence from animal and cellular studies. Most of the published research has focused on specific types of MNPs to assess their toxicity, while other types of plastic particles commonly found in the environment remain unstudied. Future studies should investigate MNPs exposure by considering realistic concentrations, dose-dependent effects, individual susceptibility, and confounding factors.

**Search strategy and selection criteria**

To identify relevant and recent studies for this review, scientific databases such as Science Direct, PubMed/Medline and Google Scholar were searched, using the following keywords: micro-and-nano plastics AND [health OR adverse effects OR toxicity, OR respiratory system OR cardiovascular system or hepatic system OR renal system, gastrointestinal system OR reproductive system, OR endocrine system OR musculoskeletal system, OR nervous system OR immune system OR metabolic effects OR other effects]. Only articles published in English were included.

## Introduction

The industrial benefits of plastics are widespread; production is expected to quadruple by 2050.^1^ However, their widespread distribution and presence in various environmental niches (air, water, or land) make humans vulnerable to exposure through multiple routes (Domenech and Marcos, 2021). Plastic materials are broken down through oxidation, hydrolytic degradation, photodegradation, mechanical degradation and biodegradation, producing various forms and sizes of debris, leading to the term “microplastic”^2^ and, more recently: plastic nanoparticles (≤100 nm), nanoplastic (100–1000 nm), microplastics (1 μm < 1000 μm), mesoplastics (0.5–5 cm), macroplastics (5–50 cm), and megaplastics (>50 cm).^3^^,^^4^

Microplastics (MPs) are added to commercial products (primary MPs), including cleaning products and fertilisers, or are produced through the degradation of larger plastic materials (secondary MPs).^5^ Nanoplastics (NPs), a subset of MPs, are typically generated either by the fragmentation of MPs or released from other sources such as plastic materials used in electronics, paints, adhesives etc.^6^^,^^7^ Secondary MPs and NPs are generally produced by the breakdown of macroplastics via shear forces,^8^ accounting for about 70–80% of total plastic released into the natural environment, while primary MPs account for 15–30%.^9^ MPs are present in different forms, such as microfibers from textiles (diapers, fleeces, and disposable masks), fragments, plastic pellets and nurdles from industry, foam, and microbeads.^10^^,^^11^ Humans are exposed to MNPs through ingestion, inhalation and skin contact,^12^^,^^13^ as described below.

### Dietary exposure

Evidence of MPs in human stool confirms their ingestion through diet.^14^ MNPs are primarily found in food, drinking water and plastic food packaging,^15^ with varied exposure levels depending on age, sex, diet and lifestyle. Wildlife species also ingest MNPs, entering the food chain and our diets,^16^^,^^17^ posing a significant threat to food safety. Due to the presence of MPs in aquatic environments, it has been detected in different types of seafood.^18^, ^19^, ^20^, ^21^ Consumption of the whole soft tissue of bivalves is a source of human exposure to MPs,^22^^,^^23^ although low levels are found in farmed bivalves from Germany (0.36 particles/g), France, Belgium and the Netherlands (0.2 particles/g).^22^^,^^24^ In Canada, 500-fold higher levels of MPs were observed in the same type of bivalves, indicating that the levels of MPs may vary by geography as well as extraction methods.^25^ A UK study predicted consumers' ingestion of 70 microplastic items in 100 g processed mussels.^26^

Table salt (1–10 MPs/kg) is another source of MPs exposure.^27^ MPs particles have also been detected in zooplanktonic organisms,^28^^,^^29^ suggesting MP could enter the food chain. Processed foods such as bottled water and milk are vulnerable to MPs introduced during processing and packaging, but their risk is unclear.^30^ In addition, MNPs in our diet can be deposited from the air, mainly indoors.^31^ MP particles that were commonly detected in foodstuffs and the environment are comprised of polyethene-terephthalate (PET), polyethene (PE), polypropylene (PP), polyvinyl chloride (PVC), polystyrene (PS), polyester (PES), polyurethane (PU), polyamide (PA), styrene acrylate and polymethyl-methacrylate (PMMA).^16^^,^^32^

### Exposure through inhalation

Inhalation is another significant route of human exposure to MNPs. The hypothesised sources of airborne MPs are synthetic textiles, construction materials, road-wear particles, abrasions of plastic materials, landfills, sewage sludge, and waste incineration.^12^^,^^33^, ^34^, ^35^ MPs were found in indoor air, constituting 4% of indoor airborne particulates.^36^ It has been estimated that, on average, an individual inhales up to 130 MPs per day.^12^ MPs can reach the human respiratory system and cause adverse health effects; industrial workers are more susceptible to exposure to MPs.^37^^,^^38^ Aerodynamic size is a defining factor in determining the depth of particle distribution within the airways once inhaled. The smaller the particle, the more likely it is to reach deeper lung regions. Inhaled particulate matter below 2.5 microns in aerodynamic size is of concern due to the higher probability of reaching the alveolar sacs, where gas exchange and particle translocation from epithelial to endothelial cells occurs.^39^

### Exposure through dermal contact

While dermal exposure is considered the least important entry route, evidence suggests that NPs can pass through the skin barrier.^13^ Atmospheric fallout of synthetic fibres and microbeads in personal care products are the major sources of dermal exposure to MNPs.^40^ However, this issue is becoming less of a concern as more and more countries are banning microbeads in personal care products and detergents.

Once ingested or inhaled, MNP particles of a bioavailable size could translocate to internal organs, dependent on their physicochemical properties, and cause harmful effects at the cellular level if above an effective internal dose.^41^ However, there is still a lack of clear understanding regarding the effective internal dose of these particles. Understanding the effective internal dose is crucial for exposure risk assessment, as it indicates a known relationship between measured physical quantities and biological effects. When determining human exposure to MPs, it is necessary to have data on the polymer types, masses, sizes, and numbers present in water, food, or air.^42^ However, studies on human exposure have not yet provided sufficient data. On the other hand, little is known about the toxicological endpoints of MPs and their relation to environmentally relevant human exposure doses.^43^ Additionally, most of the studies have primarily focused on pristine particles,^44^ which do not reflect the complexity of secondary MPs, such as those formed from different shapes, corona formation, additives, and more. This review aims to (i) discuss the potential effects of MNPs on various organ systems in the human body, (ii) identify the gaps in the existing knowledge, and (iii) provide recommendations for future research.

## Cellular uptake of MNPs

After absorption into the body, some MPs and NPs may interact with cells, dependent on several factors, such as particle size and surface properties of the particles and the biological molecules they encounter, including carbohydrates, proteins and phospholipids.^45^ Once nanoparticles interact with biological fluids and come into contact with tissues or organs, they are exposed to protein molecules, which form a ‘crown’ called a protein corona.^46^ *In vitro* studies have demonstrated that polystyrene nanoparticles coated with a protein corona facilitate higher translocation rates, influenced by the amount and composition of proteins.^47^ Moreover, protein coronas may alter the form and characteristics of nanoparticles according to the cellular environment, potentially increasing cell interactions and toxicity.^48^ Thus, there is potential for the same to happen to MNPs.

Cellular uptake of MPs and NPs can occur in several ways. Among these, endocytosis is a crucial pathway where adhesive interactions of nanoparticles or cell membrane inactive permeation occur with channel- or transporter proteins. Endocytic pathways include phagocytosis and micropinocytosis, as well as clathrin- and caveolae-mediated endocytosis.^49^, ^50^, ^51^ Previously, latex beads <200 nm in size were taken up by mammalian HeLa cells and *Caenorhabditis elegans* coelomocytes via endocytosis.^52^

Cell types influence the mode of cellular uptake, as well as the particle physicochemical properties. Polystyrene nanoparticles (120 nm) surface-modified with amidine groups can permeate rat alveolar epithelial cells via non-endocytic pathways.^53^^,^^54^ In a recent study, plastic polymer signatures (≥700 nm) were detected in the digested blood of 22 healthy volunteers.^55^ However, the particle size distribution of these polymers is unknown. Moreover, most studies used polystyrene particles; therefore, looking at the cellular uptake process of other types of MPs particles is important.

## Translocation and biodistribution of MNPs

Understanding the translocation and distribution of MNPs can be useful in assessing toxicity and health risks to organisms. There are several reviews on the ingestion, bioaccumulation, and toxicity of MNPs in organisms^56^^,^^57^; however, reviews on their transfer within organisms are limited. Numerous studies have shown the translocation and distribution of MPs in thousands of organisms, particularly aquatic animals,^58^, ^59^, ^60^ with fishes being the most widely studied species.

MNPs enter the body through the digestive tract or inhalation via gills or lungs. MNPs have been found in the digestive tracts of fish,^61^ in human lungs,^62^ blood,^63^ heart^63^ and cirrhotic liver tissue^64^ as well as in the muscle, liver, and gonads of aquatic organisms and the kidney, spleen, and placenta of mammals.^65^, ^66^, ^67^ Their accumulation in these tissues threatens animals and increases human health risks.^68^

When MNPs are ingested, a major portion of them are passed through their digestive tracts and get excreted.^69^^,^^70^ However, a small portion remains in the intestines for several days.^69^ MNPs in the gut can be internalized in cells through endocytotic processes.^60^ In the intestine, MNPs can cause damage and inflammation, entering the bloodstream, disseminating to other tissues, and persisting for prolonged periods.^71^ A study by Wick et al. (2010) using an ex vivo human placental perfusion model demonstrated that PS-NPs with diameters up to 240 nm can permeate the placental barrier and undergo transplacental transfer.^72^ Nanoparticle bioavailability after oral uptake depends on intestinal translocation. In an in vitro study, three intestinal cell models were evaluated for nanoparticle translocation.^47^ Results showed that NP size and surface chemistry affected translocation, with 50 nm nanoparticles having a higher translocation rate (up to 7.8%) than 100 nm NPs (0.8%). Overall, the transfer of MNPs is influenced by various factors, including plastic particle characteristics and animal behaviour and development.^73^ Further research is necessary to understand the health risks of MPs, including their toxicity, distribution, and accumulation in the body.

## Impact of MNPs on organ systems

**Fig. 1:**
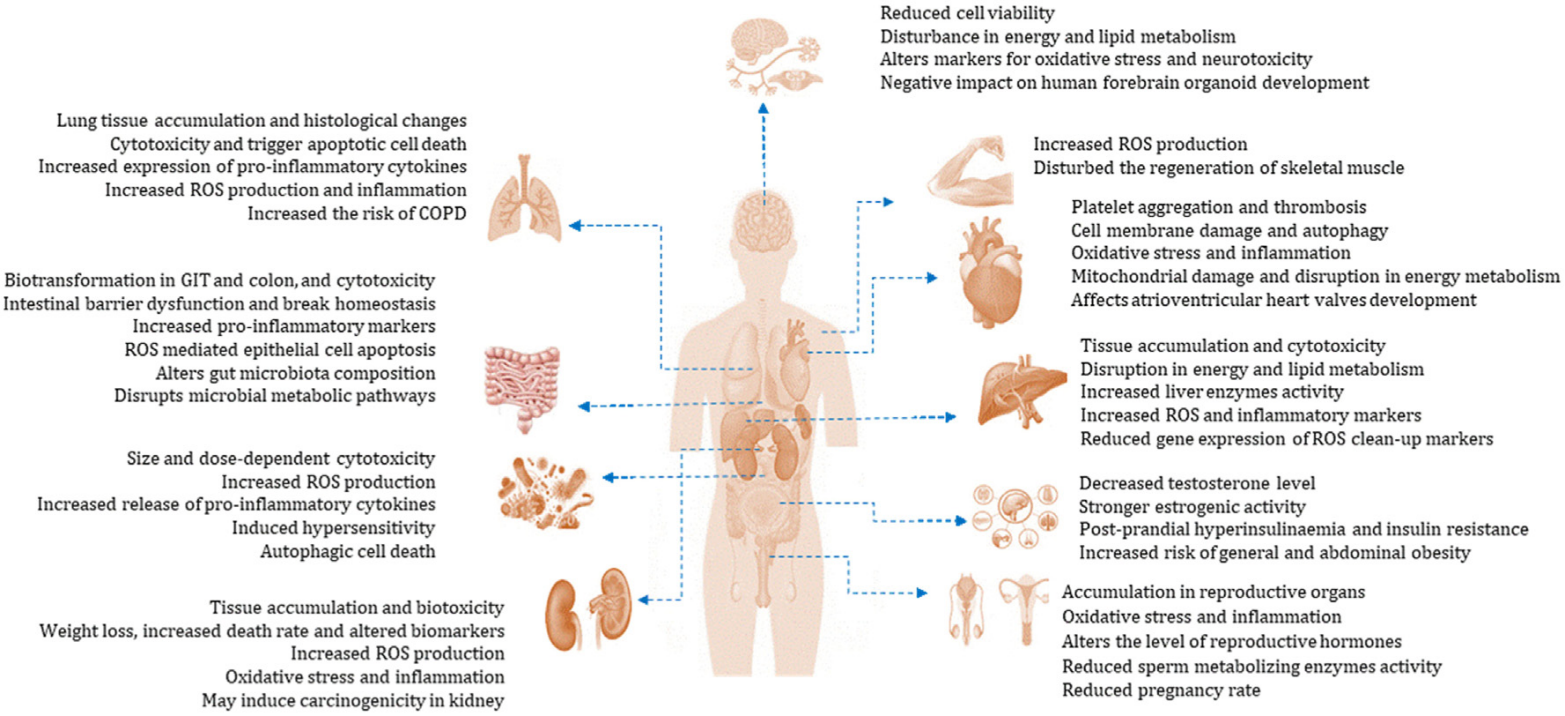
Potential effects of MNPs on different organ systems in humans.

Following exposure, bioavailable particles that enter the circulatory system can translocate to secondary organs, where they might accumulate to a level that could result in adverse effects at the cellular level. Several in vivo and in vitro studies have revealed that MNPs can have detrimental consequences. The impacts of MNPs on different organ systems have been described below, and the summary is presented in Table 1 and Fig. 1.

**Table 1:** Overview of potential effects of MNPs in various organ systems in humans and animals.

Name	Species	Study design	Particle size/exposure	Biological effects	Reference
Respiratory system	Human	Human alveolar type II epithelial A549 cell line, in vitro design	PS-NPs (25 nm and 70 nm), cells were incubated with either 1.14 μg/mL 25 nm FITC PS-NPs or 25 μg/mL 70 nm Nile red PS-NPs at different time points	• PS-NPs rapidly accumulated in A549 cells • Affect cell viability, apoptosis, and cell cycle of A549 cells • Up-regulation of pro-inflammatory cytokines (IL-8, NF-κB, and TNF-α) and pro-apoptotic proteins (DR5, caspase-3, caspase-8, caspase-9, and cytochrome *c*) • Affected gene transcription and protein expression in A549 cell	74
	Human	Human lung epithelial BEAS-2B cells, in vitro design	PS-MPs, cells were treated at 1–1000 μg/cm^2^ for 24 and 48 h	• Cytotoxicity and inflammatory effects through ROS production • May decrease transepithelial electrical resistance • May increase the risk of COPD	75
	Human	Human lung epithelial cells, BEAS-2B and human pulmonary alveolar epithelial cells, HPAEpiC	PS-NPs (40 nm), exposed to different concentrations of PS-NPs suspension for 24 h	• Reduced cell viability dose-dependently • May break redox equilibrium and cause inflammatory effects • May trigger apoptotic pathways and lead to cell death • Decreased transepithelial electrical resistance • Increased levels of matrix metallopeptidase 9 and surfactant protein A	76
	Rat	Sprague Dawley rats, in vivo design	PS-MPs (100 nm, 500 nm, 1 μm and 2.5 μm) for 3 days and intra-tracheal instillation of saline or 100 nm PS-MPs with 0, 0.5, 1 and 2 mg/200 μL for 2 weeks	• Accumulation of 100 nm and 1 μm PS-MPs in the lungs • Histological alternations were found in the lung • Increased expression of pro-inflammatory cytokines IL-6, TNF-α and IL-1β • May induce lung inflammation	77
	Mice	Transgenic mice	1-2-h inhalation of nanoparticles (Iridium and titanium dioxide NP)	• Carbon NP caused an acute and transient inflammatory response	78
Gastrointestinal system	Human	Human colon adenocarcinoma Caco-2 cell and colon adenocarcinoma HT29-MTX cell l, in vitro design	PE-MPs (1–10 μm), 14 days of daily exposure to PE MPs (21 mg of MPs in 8 mL of water, containing 0.01% (w/v)	• Increased abundance of harmful microbes (pathobionts, Enterobacteriaceae and Desulfovibrionaceae) • Decreased abundance of beneficial bacteria (Christensenellaceae and Akkermansiaceae) • Affected the composition of gut microbiota by repeated PE MPs exposure • No significant effects on mucus/epithelium barrier	79
	Human	Stimulation of GIT by combining a harmonized static model and dynamic gastrointestinal simgi model, in vitro design	PET-MPs, a single dose of digested PET MPs (0.166 g) for 72 h	• Biotransformation of PET-MPs in the GIT and colon • Structure of PET-MPs appeared to be different from the original particle • Some colonic microbiota adhere to MPs surface and promotes biofilm formation • Alters microbial colonic community composition	80
	Human	Human colon cancer cells Caco-2 and gut microbiota, in vivo cell culture design	PE-MPs and TBBPA (30–140 μm), exposed to different PEMPs concentrations (0, 100, and 1000 mg/L) for 24 and 48 h	• Co-exposure to PEMPs exacerbated the TBBPA cytotoxicity • Altered gut microbial composition and diversity • Disturbance in gut microbial metabolic pathways • Break intestinal homeostasis	81
	Mice	C57BL/6 J mice, in vivo design	PS-MPs and PS-NPs (50 nm and 500 nm, respectively), oral gavage, daily, with PS MNPs at a volume of 20 mL/kg bw for 28 days	• Intestine toxicity • Intestinal barrier dysfunction • ROS-mediated epithelial cell apoptosis	82
	Mice	C57BL/6 male mice, in vivo design	PE-MPs (10–150 μm), provided with feed containing 2, 20, and 200 μg/g MPs, for 5 weeks.	• Affected composition and diversity of gut microbiota • Increased serum pro-inflammatory cytokines (IL-1α) • Decreased Th17 and Treg cells in CD4+ cells • Induced small intestinal inflammation	83
Cardiovascular system	Human	Human embryos and hiPSCs, in vitro design	PS-NPs (40 nm and 200 nm), hiPSCs were exposed for 24 h to 1 × 10^9^/mL of 40 nm PSNPs	• Downregulation in LEFTY1 and LEFTY2, and upregulation in CA4 and OCLM genes • Affected atrioventricular heart valve development	84
	Human	RBCs, in vitro design	PS-NPs (50–250 nm), incubated 50–500 μg/mL for 1 h	• Induced hemolysis, size and dose-dependent manner in plasma-free medium but not in full plasma	85
	Human	Plasma, in vitro design	PS-NPs (57 and 330 nm), incubated 0.0–0.5 mg/mL for 15 min	• Amine-modified NPs decreased thrombin formation • carboxyl-modified NPs induced blood coagulation in plasma	86
	Human	HUVECs, in vitro cell culture design	PS-NPs (100 and 500 nm), HUVECs were exposed to 5–100 μg/mL for different periods	• 500 nm PS-NPs were bound to the surface of cell membranes • 100 nm PS-NPs were aggregated in the cytoplasm. • 25 μg/mL of 500 nm PS-NPs increased cellular release of LDH • 100 nm PS-NPs caused cell membrane damage, induced autophagy and autophagosome formation	87
	Chicken	61-day-old chicks, in vivo design	PS-MPs (5 μm), orally given at 1–100 mg/L for 6 weeks	• Pathological damage and ultrastructural changes in heart • Induced oxidative stress • Induced myocardial pyroptosis, cellular inflammation • Mitochondrial damage and disruption in energy metabolism	88
	Hamstars	Hamstars, in vivo design	PS (60 nm), intravenously and intratracheally administrated at 5–500 μg/kg	• Carboxylate-PS inhibited thrombus formation • Amine-PS increased thrombosis • Induced platelet aggregation • May affect hemostasis	89
Hepatic system	Human	Hepatocellular (Hep G2) liver cells. In vitro cell culture design	Green-fluorescent PS-MPs (1 μm), Hep G2 cells were treated with 5 μg/mL of PS-MPs for 24, 48 and 72 h	• Reduction in cell viability • Morphological changes in cells with MPs particle uptake • Reduced glycolytic activity • Increased cellular ROS production • Reduced gene expression of ROS clean-up markers (GAPDH, SOD2, and CAT)	41
	Human	hPSC-derived liver organoids, in vitro design	PS-MPs (1 μm), cells were treated at 25 μg/mL, 2.5 μg/mL and 0.25 μg/mL concentration for 48 and 72h	• Increased apoptotic cells and decreased live cells • Accumulation of harmful lipids in liver • Increased AST, ALT and LDH activity • Decreased GSH and SOD activities and increased MDA activity • Reduced ATP production • Increased ROS and inflammatory markers (IL-6 and COL1A1) levels • Increased hepatic expression of HNF4A and CYP2E1	90
	Mice	Male adult mice were divided into control and exposed group, in vivo design	Fluorescent and pristine PS-MPs (5 μm and 20 μm), treated with 0.01 mg/day to 0.5 mg/day by oral gavage for up to 28 days	• Accumulation of MPs in liver tissue • Disturbance of energy (reduction in ATP level) and lipid (reduction in TC and TG level) metabolism • Increased LDH activity • Increased GSH-Px and SOD activity and decreased CAT activity • Oxidative stress	91
	Mice	Adult male mice were divided into 4 groups, in vivo design	Fluorescent PS-MPs (5 μm), intragastrically inoculated at concentrations of 0.1, 0.5, and 1 mg/mL, respectively, for 4 weeks	• Increased AST, ALT and LDH activity • Increased levels of IL-1β, and IL-18 • Oxidative damage (increased MDA activity and decreased GSH and SOD activity) • Induced lipid peroxidation	92
	Mice	Adult male mice, in vivo design	Green fluorescent MPs (0.1 μm), orally exposed at concentrations of 0.1 mg/L and 1 mg/L for 60 days	• Induced nuclear and mitochondrial DNA damage • Increased pro-inflammatory cytokines expression • May facilitate liver fibrosis	93
	Mice	Male C57BL/6J mice were divided into control and exposed groups, in vivo design	PS-MPs (0.5 μm), administered orally for 4 weeks at 0.5 mg/day	• Up regulation in TNF-α, IFN-γ, IL-1β, IL-6 and IL-33 mRNA levels • Down-regulated in TGF-β1, IL-4, IL-5, IL-10 and IL-18 levels • Disruption in the inflammatory process via the NF-κB pathway	94
Renal system	Human	Embryonic kidney (HEK 293) cells, in vitro cell culture design	Green-fluorescent PS-MPs (1 μm), HEK 293 cells were treated with 5 μg/mL (for cell proliferation assay 100 μg/mL PS) PS-MPs for 24, 48 and 72 h	• Reduced cell viability • Morphological changes in cells with MPs particle uptake • Increased cellular ROS production • Reduced glycolytic activity • Reduced the gene expression of ROS clean-up markers (GAPDH, SOD2, and CAT)	41
	Human	Human kidney HK-2 cells, microfluidic platform, in vitro design	Fluorescent-labelled PS-NPs (50 nm), in the platform NPs enter the body through inhalation, ingestion, dermal contact and finally enter the circulatory system, thus taking place in kidneys, PS-NP perfusion (200 μg/mL) was applied for 24 h following cell culture	• MPs/NPs enter the kidney through endocytosis • Increased expression of JNK1/2/3 and TNF-α • Dysregulation of proteins related to cancer signalling, MAPK signalling (RTK, RAS, ERK, P38, NRF2, and TNF-α-R) and PI3K-AKT signalling pathways (PI3K, AKT, MDM2, P53, and ΒΑD) • May cause potential carcinogenic toxicity to kidney and testis disease	95
	Human	Human kidney proximal tubular epithelial cells, HK-2 cells, in vitro design	Fluorescent yellow-green PS-MPs (2 μm), exposed at concentrations of 0.025, 0.05, 0.1, 0.2, 0.4, or 0.8 μg/mL for 2 h or at a concentration of 0.8 μg/mL for up to 1 h	• Higher levels of ROS and mitochondrial protein Bad • Higher levels of ER stress, inflammatory markers, MitoTEMPO, LC3 and Beclin 1 • Stimulated MAPK and AKT/mTOR signalling pathways	96
	Mice	Male mice were divided into 5 groups, in vivo degin	PS-NPs (50 nm) and PS-MPs (300 nm, 600 nm and 4 μm), exposed to 5 mg with water for 24 h and 4 weeks	• Bioaccumulation and exacerbated biotoxicity • Alternations of histomorphology • Weight loss, increased death rate and altered several biomarkers • Induced oxidative stress and inflammation	97
	Mice	Male adult mice were divided into control and exposed group, in vivo design	Fluorescent and pristine PS-MPs (5 μm and 20 μm), treated with 0.01 mg/day to 0.5 mg/day by oral gavage up to 28 days	• Accumulation of MPs in kideny • Disturbance of energy and lipid metabolism • Increased GSH-Px and SOD activity and decreased CAT activity • Oxidative stress	91
Reproductive and developmental system	Human	18 mother–infant pairs from Shanghai, China, prospective pilot study, 2021	18 placentas and 12 meconium samples were collected for detection of MPs	• MPs detected in 76.5% of samples • 16 types of MPs were identified (polyamide and polyurethane were major types • Proteobacteria, Bacteroidota, and Firmicutes were the major microbiota; differences in β-diversity were also observed • Inverse relationship was observed between polyethylene and microbiota present in placenta and meconium	98
	Human	Human testis- NTE cells, microfluidic platform, in vitro design	Fluorescent-labelled PS-NPs (50 nm), PS-NP perfusion (200 μg/mL) was applied for 24 h following cell culture	• NPs enter the testis via endocytosis • Increased expression of JNK1/2/3 and TNF-α • Dysregulation of proteins in PI3K-AKT signalling pathway	95
	Mice	Male and female C57BL/6 mice, in vivo design	Pristine PS-MPs (5.0–5.9 μm), exposed at a concentration of 0.1 mg/day for 30 succinate dehydrogenase (SDH) and lactate dehydrogenase (LDH) and 44 days	• Accumulation of PS-MPs in the testis and ovary • Reduced ovary size and number of follicles • Altered reproductive hormone level • Reduced pregnancy rate • May damage testes and ovaries and induce oxidative stress	99
	Mice	Female mice, in vivo design	PS-MPs/G-PS-MPs (0.79 μm), oral gavage at 30 mg/kg for 35 days	• Accumulation of PS-MPs in uterus, ovary, blood and other organs • Reduced GSH and MMP • Increased ROS production in oocytes • Induced reproductive toxicity	98
	Mice	Male mice were divided into 8 groups,	MPs particles (5.0–5.9 μm), exposed to different doses of MPs for 6 weeks.	• Significant decrease in number and motility of sperm, and increase in sperm deformity rate • Decreased sperm metabolism-related enzymes (SDH and LDH) activity • Decreased serum testosterone level	100
Nervous system	Human	3D model of human forebrain cortical spheroids, in vitro design	PS-MPs (1 μm and 10 μm), exposed at concentration of 100, 50, and 5 μg/mL during day 4–10 and day 4–30	• Short-term exposure to MPs caused increased proliferation and gene expression of Nestin, ATF4, PAX6, SOD2and HOXB4 • Long-term exposure reduced cell viability and downregulated mature neuronal markers and cortical layer VI markers • Effects of PS-MPs on the brain were dose-dependent and based on particle size • Negative impact on human forebrain organoid development	101
	Mice	Male adult mice were divided into control and exposed group, in vivo study design	Fluorescent and pristine PS-MPs (5 μm and 20 μm), treated with 0.01 mg/day to 0.5 mg/day by oral gavage up to 28 days	• Disturbance in energy and lipid metabolism • Alters oxidative stress markers • May reduce AChE activity and cause neurotoxicity	91
Immune system	Human	Human colon (colorectal adenocarcinoma) cell line Caco-2 and HT29-MTX-E12 and human blood monocyte-derived macrophages and dendritic cells, in vitro design	MP polymer (50–500 μm), exposed at concentrations of 823.5–1380.0 μg/cm^2^	• Some changes in inflammatory cytokines levels and intestinal barrier integrity, but not statistically significant	102
	Human	Human PBMCs, HMCs-1, HDFs, and HeLa cells, in vitro design	MP derived from PVC and ABS	• Induced the release of IL-6 and TNF-α • Suppressed histamine release	103
	Human	Human PBMCs and HMC-1 cells and murine Raw 264.7 cells	PP-MPs (∼20 μm and 25–200 μm)	• Size and dose-dependent cytotoxicity • Increased ROS production • Increased release of pro-inflammatory cytokines (IL-6 and TNF-α) • Induced hypersensitivity	104
	Human and Mice	Human bronchial epithelial cell line BEAS-2B and mouse macrophage cell line RAW 264.7, in vitro cell culture design	Carboxylated and amino-modified PS-NPs (60 nm), NP solutions were prepared at 5 mg mL^−1^ and added to cell culture	• Autophagic cell death • Authophagic flux • Excess ROS production • ER stress	105
Endocrine system	Mice	BALB/C mice, in vivo design	PS-MPs (0.5 μm, 4 μm, and 10 μm), exposed at 10 mg/mL by oral gavage per day for 28 days	• Decreased testosterone level • Testicular inflammation and disruption in the blood-testis barrier	106
Muscular system	Mice	C57BL/6 male mice, in vivo design	PS-MPs (1–10 μm and 50–100 μm), exposed to different concentrations for up to 72 h	• ROS overproduction • Disturbed the regeneration of skeletal muscle	107

### Respiratory system

Inhalation is one of the main routes of human exposure to MNPs. However, it is not clear yet to what extent airborne MNPs cause adverse effects on the respiratory system. A major part of the inhaled MNPs can be cleared through a mechanical process, phagocytosis or lymphatic transport.^108^ However, some thin fibres can still be deposited in alveoli, alveolar ducts, and terminal bronchioles, leading to chronic inflammation and fibrosis.^109^^,^^110^ The severity of tissue damage typically depends on the inhaled dose over a prolonged period.^111^ Recent studies have identified MPs in human pulmonary tissue^62^ and sputum samples,^112^ indicating that inhalation is an important route for plastic particles to enter the human body.

Several studies on cell cultures have assessed the potential effects of MNP particles on human lung epithelial cells, which serve as a model for respiratory toxicity. One study by Xu et al. (2019) examined the impacts of PS-NPs particles, with 25 nm and 70 nm diameters, on the human alveolar epithelial cell line A549.^74^ The results showed that PS-NPs caused cell damage, inflammation, and apoptosis. They also triggered the up-regulation of pro-inflammatory cytokines and pro-apoptotic proteins, indicating that PS-NPs influenced the TNF-α-mediated apoptosis pathway.^74^ Increased TNF-α expression is associated with airway inflammation in asthma. In another study, PS-MPs have been found to increase inflammation and cytotoxicity in human lung cells (BEAS-2B) by increasing ROS production.^75^ Exposure to high doses of PS-MPs may increase the risk of chronic obstructive pulmonary disease (COPD), especially in individuals with lower levels of α1-antitrypsin.^75^ In another study, exposure of human lung cells (BEAS-2B and HPAEpiC) to PS-NPs caused reduced cell viability in a dose-dependent manner.^76^ PS-NPs were also found to cause redox imbalance, inflammation, apoptotic pathways, and cell death. Furthermore, the study suggested that redox imbalance could play a key role in PS-NPs-induced lung injury. Increased levels of matrix metallopeptidase 9 and Surfactant protein A were observed in PS-NPs treated cells, indicating a decrease in the lung's repair ability and potential tissue damage.^76^

In animal studies, lungs showed an increased susceptibility to inhaled nanoparticles (Iridium and titanium dioxide nanoparticles) in a murine cystic fibrosis model.^78^ In another study, transtracheal injection of PS-MPs (100 nm, 500 nm, 1 μm and 2.5 μm for 3 days) caused the expression of pro-inflammatory cytokines (TNF-α, IL-6, and IL-1β) in alveolar lavage fluid in rats.^77^

MNPs reach the respiratory epithelium and translocate via several processes, such as diffusion, cellular penetration, or cellular uptake.^113^ In the alveoli, the smallest particles (<1 μm) generally follow a diffusion process across membrane pores, whereas, for particles sized 1–3 μm, phagocytosis is the main pathway of transportation.^114^ The toxicological effect of inhaled nanoparticles depends on their interaction with the pulmonary surfactant (PS) film. In vitro and silico studies indicated that hydrophilic nanoparticles quickly move across the PS film, while hydrophobic nanoparticles get stuck and are enclosed within lipid protrusions.^115^ The study suggests a new PS lipoprotein corona model linked to inhaled nanoparticles with different physicochemical properties.^115^ Nanoparticles may also cross the surfactant at different breathing stages due to varying surface tension and lipid packing.^116^ Studies at the molecular level have shown that proper lipid packing and hydrophobic protein assembly are crucial for a healthy lung surfactant film.^117^^,^^118^ Other studies reported an interaction of PS-NPs with biological components such as proteins, termed “corona”, which appears to affect the degree of toxicity.^119^ Moreover, in the lung, PS can alter ZnO nanowire's (ZnONWs) properties, affecting their internalization and processing by epithelial cells.^120^ Overall, this suggests that inhaled MPs and NPs might differ in their level of uptake based on the molecules that bind to their surface. The above study findings indicate that MNPs inhalation may affect respiratory health through oxidative stress and inflammatory reactions, followed by epithelial barrier destruction and cellular death, resulting in tissue damage and respiratory disease after long-term exposure.

## Gastrointestinal system

Impaired gastrointestinal tract (GIT) function leads to disturbance in the regulation of energy homeostasis. Evidence shows that MPs and NPs can be accumulated easily in the GIT.^121^^,^^122^ However, the effects of MNPs on human gut microbiota, inflammation and associated mechanisms are not yet fully understood. A study by Fournier et al. (2023) evaluated the effects of Polyethylene (PE) MPs (1–10 μm) on the human gut microbiota intestinal barrier using a Mucosal Artificial Colon (M-ARCOL) model, coupled with a co-culture of Caco-2 and mucus-secreting cells.^79^ Exposure to PE-MPs (21 mg of MPs in 8 mL of water for 14 days) increased harmful microbes like pathobionts, Desulfovibrionaceae, and Enterobacteriaceae while reducing beneficial microbes like Christensenellaceae and Akkermansiaceae in terms of composition and metabolic activity. However, there were no significant effects on permeability and inflammation on the mucus/epithelium barrier.^79^ Although this study provides important information on MPs exposure effects on the human gut, the major limitation was that it only used virgin PE microbeads that are not fully representative of ingested forms based on their spherical shape.^79^ Moreover, whether the observed effect is due to a high dose of PE-MPs or any persistent particles is unclear. This could have been clarified by including another MP type or a different particle. Studies show that polyethylene terephthalate (PET)-MPs can undergo biotransformation in the human digestive tract and colon, altering the structure of the particles and the microbial community composition in the colon.^80^ A separate study indicated that PE-MPs co-exposure can exacerbate tetrabromobisphenol A (TBBPA) cytotoxicity and alter the gut microbial composition, diversity, and metabolic pathways, which may disrupt nutrient metabolism, ultimately breaking gut homeostasis.^81^ Genes encoding plastic-degrading enzymes were found in the human gut microbiome, indicating an adaptation of the gut microbiome to MPs pollution (Nugrahapraja et al., 2022).

In animal studies, MP exposure has also been shown to cause adverse effects on the intestinal system. Combined exposure to PS-NPs and PS-MPs (50 nm and 500 nm, respectively, at a concentration of 20 mL/kg bw for 28 days) caused intestinal barrier dysfunction by epithelial cell apoptosis though ROS production in mice model.^82^ In another study in mice model, exposure to PE-MPs (10–150 μm) at various concentrations (2, 20, and 200 μg/g for 5 weeks) showed more gut microbial species, increased bacterial abundance, flora diversity and increased pro-inflammatory cytokines secretion.^83^ The study's results suggest that exposure to PE-MPs can cause intestinal dysbacteriosis and inflammation via the TLR4 signalling pathway. The gut microbiota is vital for evolution, metabolism, immune defence, and disease susceptibility^123^; therefore, The potential effects of MNPs on the human gut must be studied further.

## Cardiovascular system

If they enter the bloodstream, MNPs may also cause adverse effects on the cardiovascular system. However, there is limited information on the cardiovascular effects of MNPs exposure. Recent evidence shows that MPs in multiple human heart tissues and blood.^63^ A human cell-based investigation indicated that the development of atrioventricular heart valves was affected remarkably after exposure of PS-NPs (40 nm and 200 nm, at a concentration of 1 × 10^9^/mL) to human-induced pluripotent stem cells (hiPSCs) for 24 h.^84^ The migration of MNPs through the bloodstream to the heart has been reported in mice and rats.^82^^,^^98^^,^^124^ For example, oral exposure (5 and 50 mg/L) of Wister rats to PS-NPs (0.5 μm) for three months showed the accumulation of NPs in the myocardial cells.^124^ The translocation of MNPs to the heart depends on particle size and surface modification.^47^ When the particle size is 460 nm and 1 μm may affect the RBCs.^125^

MNPs may affect cardiac functions and cause toxicity on (micro) vascular sites.^126^ In zebrafish embryos, environmentally relevant concentrations of PS-MPs and PP-MPS caused reduced heart rates.^127^ However, the associated mechanisms are not precise yet. Some possible mechanisms include the direct interaction of MNP with cardiac sarcomeres and subsequent effects on the heart rate^114^ or the indirect induction of ROS-mediated oxidative damage by MNP exposure that leads to heart cell damage, affected blood circulation and impaired heart function.^128^^,^^129^ In addition to heart rate, attention should also be given to heart rate variability (HRV), as lower HRV is associated with hypertension and atherosclerosis.^130^^,^^131^

Apart from heart rate, some studies indicated that MNP exposure could affect cardiac functions. In a study, the exposure of rats to PS-MPs (0.5 mm, 0.5–50 mg/L) caused damaging the heart's structure and function with impaired mitochondrial intregity and increased creatine kinase-MB and cardiac troponin I levels.^132^ It has also been suggested that PS-MPs-induced oxidative stress promotes cardiomyocyte apoptosis and leads to cardiac fibrosis through activation of the Wnt/β-catenin pathway in rat models.^124^ However, the major limitation of this two studies is that it used monodisperse polystyrene spherical particles. Therefore, it is unclear how to extrapolate these results to environmentally relevant exposures. In another study, MNPs has been shown to affect the cardiac cells through oxidative stress, inflammation, apoptosis, and pyroptosis. Oral exposure of chicken to PS-MPs (5 μm; 1–100 mg/L) for six weeks caused myocardial fibre damage, cellular inflammation, and mitochondrial lesions.^88^

MNPs can cause micro-vascular toxicity through hemolysis, thrombosis, blood coagulation and damaging of endothelial cells. There is evidence that MNPs can cause hemolysis in human cells in vitro. For example, in a protein-free medium, treating human red blood cells (RBCs) with PS-NPs (50–250 nm; 50–500 μg/mL) caused significant hemolysis, and the degree of hemolysis was particle size and dose-dependent.^85^ Thrombosis is a considerable risk factor for cardiovascular disease. In animal studies, exposure of hamsters to PS particles (60 nm; 5–500 μg/kg) with surface modifications caused different thrombotic events.^89^ The study showed that unmodified particles did not exert effects on thrombosis up to 5 mg/kg bw. In contrast, carboxylate-polystyrene particles inhibited thrombus formation at 500 and 100 μg/kg bw but not at 50 μg/kg bw. On the other hand, amine-polystyrene caused thrombosis at 50 and 500 μg/kg bw but not at 5 μg/kg bw.^89^ Exposure to MNPs can impact the equilibrium between pro- and anticoagulant pathways. In human plasma, amine-PS-NPs (57 and 330 nm; 0.06–0.5 mg/mL) showed reduced production of thrombin and induced blood coagulation.^86^ Exposure of human umbilical vein endothelial cells (HUVECs) with PS-NPs (100 and 500 nm; 5–100 μg/mL) showed to interact with the endothelial cell membrane of the human umbilical vein, caused endothelial damage, induced autophagosome formation and autophagic flux blockage in endothelial cells.^87^ Overall, vascular endothelial damage by MNPs may lead to a series of cardiovascular events through disruption in endothelial growth factor, the interaction between endothelial cells and blood and immune cells, endothelial dysfunction and inflammatory responses.

## Hepatic system

The liver is the primary organ that is affected by various xenobiotic substances, including MNPs.^133^ Impaired liver function can affect many other organs in the body. Up to now, only a few studies have evaluated the effects of MNPs on the liver. To determine the presence of MPs, the research examined liver tissue samples from patients with liver cirrhosis and participants without underlying liver disease.^64^ The study found six types of MPs polymers only in cirrhotic hepatic tissue samples, suggesting that chronic liver disease may be a key driver in MP accumulation in the liver.^64^ However, the exact role of MPs in liver disease and their relation to hepatic fibrogenesis and cirrhosis development is not clear.^64^

In an in vitro study, human hepatocytes (Hep G2) were used to assess the toxicological impact of polystyrene MPs (PS-MPs, 1 μm) at various concentrations.^41^ Exposure to PS-MPs (100 μg/mL) caused a significant decrease in cell proliferation but no significant reduction in cell viability. Furthermore, qRT-PCR analysis indicated that gene expression reduced for glyceraldehyde-3-phosphate dehydrogenase (GAPDH), an enzyme involved in glycolysis, and antioxidant enzymes catalase (CAT) and superoxide dismutase 2 (SOD2), thus decreasing the potential of SOD2 and CAT in detoxifying ROS. The overall results of this study suggest that 1 um PS-MPs of 100 μg/mL reach the liver and may lead to alteration in cell–cell interactions and cell metabolism. In another in vitro study, liver organoids (LOs) produced from human pluripotent stem cells (hPSC) were exposed to different concentrations of 1 μm PS-MPs (0.25, 2.5 and 25 μg/mL PS-MP).^90^ The results showed that PS-MPs, even at the lowest concentration, could induce hepatotoxicity and lipotoxicity through cytotoxicity (increased apoptotic cells and decreased live cells) and alter molecular markers (increased AST, ALT and LDH activity in supernatants, decreased GST activity, GSH and SOD contents in the LOs, and increased MDA contents indicative of oxidative stress). PS-MPs caused lipid accumulation in LOs, reduced ATP production, increased ROS production, and released inflammatory markers IL-6 and COL1A1. PS-MPs also increased hepatic HNF4A and CYP2E1 expression, increasing the risk of liver steatosis and fibrosis.^90^

A number of in vivo studies evaluated the effects of MNPs on the liver. For example, fluorescent PS-MPs (5 μm and 20 μm) were administered to male adult mice in varying doses (0.01–0.5 mg/day).^91^ Treated mice showed decreased liver weight, accumulation of PS-MPs in liver tissue, and inflammation and lipid droplets in the liver. The exposed mice also showed reduced ATP levels, increased LDH activity, and lowered TC and TG levels. This suggests that PS-MPs exposure may impact energy metabolism and liver function. However, the study's major limitations are the difficulties of interpreting the finding of adversity of alterations of intermediary metabolite levels, especially the images that don't support the claims, and MP concentrations in the liver exceed the delivered dose.^134^ Male mice exposed to green fluorescent MPs orally for 60 days showed that even at the lower concentration of 0.1 mg/L, MPs can cause damage to both nucleus and mitochondrial DNA, releasing double-stranded DNA fragments into the cytoplasm.^93^ This triggers the activation of the STING pathway, leading to liver fibrosis.^93^ In further mouse study, oral administration of PS-MPs (0.5 μm) for 4 weeks at 0.5 mg/day increased liver weights and function parameters and caused infiltration of NK cells and macrophages while reducing that of B cells.^94^ In a study, male mice were exposed to 5 μm fluorescent PP-MPs (0.1, 0.5, and 1 mg/mL) for four weeks.^92^ Results showed that PP-MPs can damage the liver by breaking and reducing mitochondrial cristae, increasing the activity of liver enzymes and causing pyroptosis, oxidative damage, and lipid peroxidation.^92^ Abdel-Zaher et al. (2023) study found that PE-MPs exposure (100 nm, 600 μg/day) for 15 days impacted the liver function in a murine model.^135^ Further, PE microbeads (36 and 116 μm) ingestion (100 μg/g of food for 6 and 9 weeks) showed exacerbated liver fibrogenesis in mice.^136^ Most studies have focused on MNPs' inflammatory and oxidative stress-mediated liver injury. Therefore, future studies should explore other possible mechanisms of MNPs-induced liver damage.

## Renal system

Impairment of kidney function can result in the accumulation of toxins and impurities in the bloodstream, which can adversely affect overall health. In a study MPs (PVA, PVC, PP and PE, 4–15 μm size) were found in human urine, suggesting that they can pass through the gastrointestinal tract and be excreted through biological processes.^137^ A review by Prata et al. (2023) indicated that MNPs are mainly excreted by the liver via phagocytosis or biliary excretion in faeces and the kidney via biodegradation products in urine.^138^

Some cell cultures and animal studies determined the potential effects of MNPs on kidneys. In a study by Goodman et al. (2022), human embryonic kidney cells (HEK 293) were exposed to 1 μm PS-MPs (5 μg/mL for up to 72 h).^41^ The results showed that PS-MPs caused significant morphological changes in the kidney cells, uptake of PS-MP particles, and reduced cell proliferation. The exposed cells observed increased ROS levels.^41^ In another study, researchers exposed human kidney (HK-2) and testis (NTE) cells to fluorescent-labelled PS-NPs (50 nm, 200 μg/mL for 24 h) and found that the PS-NPs can enter the cells through endocytosis, causing damage to cellular microstructures and an increase in the expression of JNK1/2/3 and TNF-α.^95^ Wang et al. (2021) explored the effects of PS-MPs (2 μm) on human HK-2 cells.^96^ The study revealed that PS-MP exposure (0.025–0.8 μg/mL for 2 h), caused an increase in ROS and mitochondrial protein Bad levels, as well as ER stress, inflammatory markers, and proteins LC3 and Beclin 1. Furthermore, PS-MPs impacted the MAPK and AKT/mTOR signalling pathways.^96^

Several studies have investigated the impact of MNPs on kidneys in animals. One such study conducted by Meng et al. (2022) involved the use of PS-MPs (300 nm, 600 nm, 4 μm) and PS-NPs (50 nm) on male mice at a concentration of 5 mg with water for 24 h and 4 weeks, resulting in weight loss, increased death rates, altered biomarkers, and histological damage to the kidney.^97^ The study also found bioaccumulation of MPs and NPs in the kidneys, with the 600 nm particles causing exacerbated toxicity. Moreover, PS-NPs and PS-MPs cause oxidative stress and inflammation. In a further study, mice were exposed to various concentrations of 5 μm and 20 μm fluorescent PS-MPs, and the results indicated a strong fluorescence expression in the kidneys and disturbance in lipid metabolism.^91^ However, the study had some limitations mentioned in the hepatic system section.

## Reproductive and developmental system

Some animal studies have reported on the reproductive and developmental toxicity of MNPs,^99^^,^^100^ but still, there is limited information for humans. A study in China found 16 types of MPs in placentas and meconium samples, with polyamide and polyurethane being the major types.^139^ MPs were detected in about 76.5% of the samples with a size of 20–50 μm. The major microbiota found were Proteobacteria, Bacteroidota, and Firmicutes. The study also indicated an inverse correlation between polyethylene and the Chao index of meconium microbiota and several genera of placenta microbiota.^139^ Xiao et al. (2023) found that PS-NPs (50 nm) can enter testis cells through endocytosis and impact micro-structures.^95^ Dysregulated proteins were found on cancer signalling pathways such as PI3K-AKT and MAPK. The activated PI3K-AKT pathway plays a significant role in sperm cell proliferation, survival, and testicular growth.^95^ Moreover, it facilitates the interaction between FSH and support cells, which helps in maintaining testicular stability in males.^140^

Studies have shown that exposure to PS-MPs can cause reproductive toxicity in mice. For example, exposure to PS-MPs (5.0–5.9 μm) in male-female mice caused testis and ovarian damage, oxidative stress, hormonal changes, and reproductive issues.^99^ Furthermore, decreased pregnancy rate and fewer embryos were observed after PS-MPs exposure. Female mice showed greater susceptibility to MPs exposure than male mice.^99^ In female mice, PS-MPs exposure was shown to cause inflammation of ovaries and decrease the quality of oocytes in mice, which indicates reproductive toxicity.^98^ In a separate study, male mice exposed to PS-MPs (5.0–5.9 μm) in saline solution for six weeks had reduced sperm count and motility, increased sperm deformity rate, and lower serum testosterone levels. MNPs may also disrupt energy balance and cause reproductive toxicity. However, it’s unclear if the effects on testicular function are directly caused by exposure to the test materials since testicular histology wasn't analyzed.^141^ The effects on sperm may also be due to epididymis defects.

## Nervous system

The central nervous system in humans is highly susceptible to environmental pollutants, especially during embryonic development.^142^ Therefore, Exposure to micro- and nanoplastics can induce oxidative stress, which may cause cellular damage and increase vulnerability to neuronal disorders.^143^ Due to their size, MNPs can cause stronger neurotoxicity than MPs.^144^ Exposure to MPs can affect the central nervous system by influencing acetylcholine, γ-aminobutyric acid, and glutamate.^145^ Impaired AChE activity can lead to excessive accumulation of acetylcholine, triggering neurological disorders.^146^

Hua et al. (2022) studied the effects of PS-MPs (1 μm and 10 μm, 5, 50 and 100 μg/mL for 4–10 days and 4–30 days) on the human brain using a 3D model of cortical spheroids.^101^ Short-term exposure promoted cell proliferation and gene expression of Nestin, ATF4, PAX6, SOD2, and HOXB4, but long-term exposure decreased cell viability. PS-MPs also affected gene expression of DNA damage and neural tissue patterning.^101^ Neural progenitor cells are likely to uptake and internalize MP 1 μm through processes such as phagocytosis or endocytosis.^147^

In an animal study, male mice were orally exposed to fluorescent PS-MPs (5 μm and 20 μm) showed reduced AchE activity,^91^ altering cholinergic neurotransmission efficiency,^148^ and causing neurotoxicity and oxidative stress.^91^ MPs exposure caused the elevation of serum levels of threonine, aspartate and taurine while reducing phenylalanine, a precursor to neurotransmitters. Despite insufficient evidence, further investigation is needed due to the potential risks of MNPs on the nervous system.

## Immune system

Exposure to toxins may induce local or systemic immune reactions based on their dissemination. However, in the case of genetic susceptibility, only environmental exposure may impair the normal function of the immune system, favouring immunosuppression or autoimmune diseases.^149^

Upon MP exposure, immune cells cause strong modulation at the transcriptional level of enzyme levels to the release of cytokines. When MPs are exposed, immune cells cause significant changes in various transcriptional levels, including enzyme levels and the release of cytokines. A study conducted by Lehner et al. (2020) developed an in vitro 3D intestinal model comprising human intestinal epithelial cell lines Caco-2 and HT29-MTX-E12, along with human dendritic cells and blood monocyte-derived macrophages to investigate the potential effects of ingested MPs, such as 50–500 μm MP polymer representing tire wear and polyolefins at concentration 823.5–1380.0 μg/cm2.^102^ Although the results showed some changes in inflammatory cytokine levels (IL-8, TNFα, and IL-1β) and the barrier integrity, these changes were not significant.^102^ In another in vitro study, human bronchial epithelial cells (BEAS-2B) and mouse macrophages (RAW 264.7) were exposed to 60 nm PS-NPs, and showed high toxicity and autophagic cell death. NH2-PS caused autophagic flux and activated the autophagic cell death process via Akt/mTOR and AMPK pathways.^105^ Han et al. (2020) found that exposure to polyvinyl chloride (PVC) and acrylonitrile butadiene styrene (ABS) for 4–5 days caused an immune response in human immune cells.^103^ The study showed that both PVC and ABS suppressed histamine release while increasing the release of IL-6 and TNF-α. However, long-term exposure could lead to a higher immune response, which the study has not investigated.^103^ Other form of MPs, polypropylene MPs (PP-MPs, size ∼20 μm and 25–200 μm) were shown to induce local immune responses by triggering the production of pro-inflammatory cytokines such as IL-6, TNF alpha, and histamine in a size and concentration-dependent manner.^104^

In the mice model, high concentrations (600 μg/day) of PE-MPs (10–150 μm) altered the diversity and composition of intestinal microflora and caused inflammatory reactions through increased expression of TLR4, AP-1, and IRF5.^83^ The serum level of IL-1α was also elevated significantly, and Th17 and Treg cells in CD4+ T cells were decreased after MPs exposure.^83^ While there is some evidence of the effects of MNPs on the immune system, most of the studies have focused solely on the innate immune response. The impact of MNPs on the adaptive immune response remains poorly understood.

## Endocrine system

Various components found in MPs, such as Bisphenol A (BPA) and phthalates, are considered endocrine-disrupting compounds (EDCs). These EDCs can potentially impair the endocrine system's normal function. EDCs can enter the body and affect hormonal balance as agonists or antagonists, causing neuroendocrine effects during critical developmental stages like the perinatal period.^150^ A study demonstrated that PS-MPs (0.1 g/L) can alter the decabrominated diphenyl ether (BDE-209) degradation pathways and enhance the toxicity of the endocrine system and thyroid gland in aquatic organisms.^151^ In an in vivo study, a single dose of BPA (10 μg⁄kg) caused a rapid elevation in plasma insulin and consequently reduced glycaemia.^152^ Reports have shown that BPA with other EDCs, such as phthalates,^153^^,^^154^ may increase the risk of developing type 2 diabetes. A study by Jin et al. (2021) reported that PS-MP (0.5 μm, 4 μm, and 10 μm) exposure (10 mg/mL, via oral gavage for 28 days) can decline testosterone levels in male mice, cause testicular inflammation, and disrupt the blood-testis barrier.^106^

## Muscular system

Compared to other organs, few studies have assessed the effects of MNPs on the muscular system. Until now, there is no direct evidence of the accumulation of MNPs in human muscle tissue. A study indicated the accumulation of MPs in the muscle tissue of northern fulmars due to the consumption of fish^155^^,^^156^ and other seafood.^157^ However, no significant correlation with muscle damage was observed.^155^^,^^156^ A study in a mouse model showed that PS-MPs (1–10 μm and 50–100 μm) exposure disrupted muscle fibre regeneration and the balance between myogenic and adipogenic differentiation.^107^ Furthermore, PS-MPs exposure induced the overproduction of ROS in satellite cells and altered the p38 MAPK and NF-κB pathways.^107^

## Other effects

In addition to specific organ effects, MNPs can cause biochemical, metabolic, genotoxic, and carcinogenic effects. As discussed earlier, MNPs can cause intestinal inflammation and liver dysfunction. However, it is not well understood whether damage and inflammation in the gut and liver may lead to severe disease development. In a recent study, mice treated with small MPs (1 μm in diameter, 10,000 μg/L in drinking water) showed an increased level of fasting blood glucose and insulin, suggesting a crosstalk between the liver and gut metabolism, which caused insulin resistance and led to the development of diabetes.^158^ These results suggest a more extensive cohort study to assess insulin resistance after MPs exposure. In a separate study, Hs27 cell lines derived from the foreskin were exposed to spherical PS-NPs (100 nm).^159^ The results showed that DNA damage was induced, resulting in an increased formation of nuclear buds and micronuclei.^159^

## Toxicity mechanisms of MNPs

The possible toxicity mechanisms of MNPs include cell membrane disruption, cell pore hindrance, ROS production, DNA damage, lysosome destabilization, and mitochondrial depolarization.^160^ Upon entering the body, MNPs first come into contact with the lungs and GIT systems, encounter biological barriers, translocate to multiple organs, and cause systemic toxicities. MNPs toxicity mechanisms mainly depend on the physiochemical properties of the particle and exposed cell types.^105^^,^^161^^,^^162^

Of different plastics, polystyrene is extensively studied and is functionalized with either positive (e.g., NH2) or negative (e.g., COOH) surface groups and shows some variation in toxicity. NH2-functionalized PS-NPs are commonly used for drug delivery and can cross the blood–brain barrier.^163^ Positive charges of NH2 reduce lipid layer thickness, making it easier for PS-NH2 to enter cells.^164^^,^^165^ In some cases, protein coronation by blood serum/digestive fluids can protect cells from damage and affect macrophage-mediated cellular uptake.^164^^,^^166^ Studies demonstrated that PS-NH2 nanoparticles of size ≤500 nm are rapidly internalized in various cells using energy-related mechanisms like micropinocytosis and phagocytosis.^17^ When NH2-PS-NP gets internalized in lysosomes, the NH2 group acts as a “proton sponge” that hinders the lysosomal compartment acidification. This leads to excessive proton pump activity and the influx of ions and water into the lysosome, causing the lysosomal membrane to dilate and suffer damage.^162^^,^^167^^,^^168^ Ultimately, the contents of the lysosome are released into the cytosol, leading to ROS and lysosomal proteases cathepsins mediated damage that cause apoptotic cell death.^162^^,^^167^^,^^168^ ROS-mediated damage can also induce autophagic cell death.^105^ These adverse effects may occur rapidly at low concentrations (5–20 μg mL−1) in various cell types such as HeLa, fibroblast lung, and intestinal cells.^105^^,^^169^ Regardless size of the particle, the surface NH2 may also play a potential role in cellular death; however, PS-NH2 particles with ≥600 nm have been shown to cause low toxicity after cellular internalization.^170^

PS MNPs, such as anionic unmodified or carboxyl-functionalized PS-MNPs, can cause toxicity.^171^ Anionic plastic particles ≤200 nm are typically internalized through endocytosis by hepatic, lung, intestinal and macrophages.^74^^,^^166^^,^^172^^,^^173^ On the other hand, anionic PS with ≤10 μm in size are normally localized to lysosomes,^105^^,^^174^ but particles with 50 nm in size trend to localize in the intestine basolateral cells nuclei^172^ and 20 nm PS-COOH localize to hepatic cells mitochondria.^173^

Unlike cationic PS, anionic PS does not destabilize lysosomes significantly, except for slight dilation and reduced enzyme activity at 40 μg/mL of 20 nm PS-COOH.^174^^,^^175^ Lower concentrations of PS have been found to cause markers of cellular stress in different cell types, such as excess ROS generation in brain (at 0.05 μg mL-1) and HeLa cells (at 10 μg mL-1)^176^ and upon PS 500 nm- 6 μm exposure mitochondrial depolarization in Caco-2 cells.^177^^,^^178^

PS-MNPs cause inflammation in the intestine, lung, and brain.^74^^,^^75^^,^^102^^,^^166^ These particles can also cause genotoxicity and excess ROS production-related DNA damage in some cells.^166^ Anionic PS-NPs can form a multi-layer protein corona that can alter protein conformation and increase the cytotoxic and genotoxic effects in mammalian blood cells.^179^ The protein corona may also significantly release pro-inflammatory proteins like IL-8 and MCP-1.^164^^,^^180^ In contrast to PS-MNPs, research on other types of MNPs, such as polypropylene and polyethylene MPs, is limited.^176^^,^^181^

In animal studies, some identical toxicities and related mechanisms were noticed as observed in humans. For example, 500 nm PS NPs at ≥25 μg mL-1 showed increased embryotoxicity, while 50 nm PS NPs at 100 μg mL-1 did not cause toxicity on fibroblast cells in mice.^182^ In invertebrate animal models, exposure to PS-NPs resulted in similar effects like excess ROS generation and cellular death.^183^, ^184^, ^185^ For example, PS-NPs exposure led to excess ROS generation and cellular death in various animal models. PET-NPs were found to disrupt mitochondrial integrity and related pathways in zebrafish embryos. Further research is required to understand the long-term effects of MNPs exposure on organs, especially in humans. Despite evidence of MNPs toxicity, in vivo, toxicity data is lacking, particularly in humans. Further research is needed to understand cellular, tissue-based, and long-term organ effects.

## Outstanding questions

• Despite indications of the harmful effects of MNPs on human organ systems, several knowledge gaps remain. For example, there is currently a lack of standardized methods for defining and detecting MPs and NPs. Therefore, it is necessary to establish a unique definition and classification of plastic debris, taking into account several criteria such as size, shape, physical and chemical properties, and sources. In addition, standard methods and sampling procedures need to be developed to detect and quantify MPs and NPs in biological specimens.
• Toxicological studies use plastics that differ from their realistic presence in the environment, making it challenging to get accurate results. Research mainly focuses on polystyrene MPs, but other forms of MPs are also present in the environment. Future studies should consider other forms of MPs and environmentally relevant concentrations to determine potential health risks.
• It remains unclear which route of exposure poses the most significant risk for humans regarding MNPs. Additionally, the elimination process of MNPs from the human body lacks sufficient information. Furthermore, it is important to investigate whether MNPs have an affinity to interact with specific chemicals or microorganisms during human exposure.
• The toxicity mechanisms of MNPs in humans are not fully understood, including their interactions with other pollutants. The long-term effects of MNPs on human health are unclear, with individual susceptibility unknown. In addition, the specific species toxicity of MNPs exposure is poorly understood. Therefore, human health risk assessment is limited and challenging. Animal and cell studies can help understand adverse effects, while observational and biomarker-based studies are needed to get deep insights. To ensure accurate observational studies, it is crucial to adjust potential confounders that are known such as such as age, sex, occupation, living place, nutritional status, and comorbidity. To achieve this, both the exposed and unexposed groups should be selected from the same source population. Furthermore, obtaining a large sample is essential to ensure the findings are representative and can be generalized to a larger population. Additionally, the investigator may choose to examine multiple outcomes simultaneously. Moreover, Future studies should integrate multi-disciplinary approaches to address this issue.

## Conclusions

MNPs are ubiquitous in the environment, and humans are frequently exposed to MNPs from multiple sources. The growing evidence suggests that exposure to MPs and NPs may cause adverse effects in different human organ systems. The available literature summarized here indicates that MNPs exposure may lead to oxidative stress, inflammation, impaired immune function, alteration in cellular and energy metabolism, inhibition in cell proliferation, tissue degeneration, abnormal organ development and dysfunction, alteration in biochemical parameters and even cause genotoxicity and carcinogenicity. Although numerous animal and cell culture studies indicated the adverse biological effects of MNPs on human health, the underlying mechanisms are still unclear. Furthermore, whether long-term exposure to MNPs is associated with disease susceptibility needs to be investigated. Further observational studies are necessary to investigate the potential adverse health consequences of MNPs in humans and the related mechanisms. Additionally, it is crucial to quantify the impact of MNPs on human health and their pathogenesis in future studies. This will help to summarize the current knowledge and address any research gaps.

## Contributors

N.A., contributed to the conceptualization, data curation, writing and editing of the manuscript. J.K., E.L.M., and T.W.G. reviewed and edited some sections of the manuscript. S.W and J.B.d.l.S. contributed by critically reviewing and editing the manuscript. All authors read and approved the final version of the manuscript.

## Declaration of interests

The authors have no conflict of interest to declare.
